# *DreamOn:* a data augmentation strategy to narrow the robustness gap between expert radiologists and deep learning classifiers

**DOI:** 10.3389/fradi.2024.1420545

**Published:** 2024-12-19

**Authors:** Luc Lerch, Lukas S. Huber, Amith Kamath, Alexander Pöllinger, Aurélie Pahud de Mortanges, Verena C. Obmann, Florian Dammann, Walter Senn, Mauricio Reyes

**Affiliations:** ^1^ Medical Image Analysis Group, ARTORG Centre for Biomedical Research, University of Bern, Bern, Switzerland; ^2^ Computational Neuroscience Group, Department of Physiology, University of Bern, Bern, Switzerland; ^3^ Cognition, Perception and Research Methods, Department of Psychology, University of Bern, Bern, Switzerland; ^4^ Neural Information Processing Group, Department of Computer Science, University of Tübingen, Tübingen, Germany; ^5^ Department of Diagnostic, Interventional, and Pediatric Radiology, Inselspital Bern, University of Bern, Bern, Switzerland; ^6^ Center for Artificial Intelligence in Medicine, University of Bern, Bern, Switzerland; ^7^ ARTORG Center for Biomedical Engineering Research, University of Bern, Bern, Switzerland; ^8^ Department of Radiation Oncology, University Hospital Bern, University of Bern, Bern, Switzerland

**Keywords:** deep learning, robustness, ultrasound, breast cancer, generative adversarial network, convolutional neural network

## Abstract

**Purpose:**

Successful performance of deep learning models for medical image analysis is highly dependent on the quality of the images being analysed. Factors like differences in imaging equipment and calibration, as well as patient-specific factors such as movements or biological variability (e.g., tissue density), lead to a large variability in the quality of obtained medical images. Consequently, robustness against the presence of noise is a crucial factor for the application of deep learning models in clinical contexts.

**Materials and methods:**

We evaluate the effect of various data augmentation strategies on the robustness of a ResNet-18 trained to classify breast ultrasound images and benchmark the performance against trained human radiologists. Additionally, we introduce *DreamOn*, a novel, biologically inspired data augmentation strategy for medical image analysis. DreamOn is based on a conditional generative adversarial network (GAN) to generate REM-dream-inspired interpolations of training images.

**Results:**

We find that while available data augmentation approaches substantially improve robustness compared to models trained without any data augmentation, radiologists outperform models on noisy images. Using DreamOn data augmentation, we obtain a substantial improvement in robustness in the high noise regime.

**Conclusions:**

We show that REM-dream-inspired conditional GAN-based data augmentation is a promising approach to improving deep learning model robustness against noise perturbations in medical imaging. Additionally, we highlight a gap in robustness between deep learning models and human experts, emphasizing the imperative for ongoing developments in AI to match human diagnostic expertise.

## 1 Introduction

One of the major factors for reduced cancer mortality is early detection through imaging-based screening (1). Recently, deep learning (DL) based methodologies have been employed to screen medical images (2–7). DL-based screening tools have not only been shown to feature high classification accuracy (8) and consistency (9) but also potentially allow for scalability: automated analysis of medical images significantly speeds up the diagnostic process and thus renders it possible to scan larger populations or conduct real-time assessments, thereby aiding in timely procedures. However, DL-based image analysis requires extensive training datasets and has been shown to suffer from generalization issues under distribution shifts (10–12). These limitations are particularly problematic in medical contexts where training data often is scarce and the need for robust generalization is critical due to the substantial variability in image quality encountered in real-world settings.

Acquiring training data for supervised learning in medical image analysis poses significant challenges including stringent privacy regulations, the need for expert annotation, as well as ensuring that the dataset represents the breadth of pathological conditions and demographic variations. Furthermore, once models are trained, learned representations must be generalized to account for the considerable variability in medical image quality, which can be affected by diverse factors such as the technical specifications and calibration of imaging devices across different healthcare facilities but also patient-specific factors. Anatomical variations across individuals, along with involuntary movement during image capture, induce an additional source of noise. Consequently, ensuring robustness against distribution shifts is essential for the successful integration of DL models into the clinical environment (13).

A distribution shift occurs when a classifier encounters an out-of-distribution test dataset whose statistical properties differ from those of the training data, posing challenges to the model's ability to generalize across new, unseen conditions. In other words, classification performance can deteriorate sharply, as the learned representations may overfit to the specific features present in the training data (10, 14). In clinical settings, this can lead to a higher rate of misdiagnoses, missed findings, or false positives. In contrast, the human visual system exhibits remarkable robustness to variations in image quality, noise, and other distortions and can therefore maintain high recognition accuracy even under challenging conditions (10, 15–17).

In deep learning, a common practice to address such generalization challenges is the use of data augmentation strategies, whereby additional synthetic data is generated by applying transformations to existing images—such as rotation, scaling, and flipping, or by simulating common artifacts and variations [for reviews on data augmentation techniques used in medical imaging see (18, 19)]. This approach helps in creating a more diverse dataset that mimics a wider array of real-world conditions without the need for extensive new data collection [e.g., see (20)]. By incorporating augmented data, DL models can be trained to be more resilient to the natural inconsistencies and discrepancies found in medical imaging. Although several reviews on the effects of data augmentation in medical imagery exist (18, 19, 21, 22), to the best of our knowledge, no systematic investigation has addressed how these strategies impact robustness to distribution shifts. Understanding the robustness impacts of specific data augmentation strategies is key to ensuring that deep learning models can reliably adapt to the diverse and unpredictable conditions encountered in clinical practice.

Here, we evaluate the robustness of three common data augmentation strategies to distribution shifts introduced by different types of parametric noise. The chosen data augmentations range from basic transformations to more complex strategies. Simple augmentations include rotation, flipping, and brightness & contrast adjustments, which provide varied versions of the original images. More advanced methods include Pixel-space Mixup (23), and Manifold Mixup (24). Pixel-space Mixup creates new training samples by blending pairs of images and their labels directly in pixel space, helping the model learn smoother decision boundaries. Manifold Mixup, extends this concept further by blending representations in deeper network layers rather than raw pixel data, thereby introducing intermediate states at a feature level. Additionally, to determine how well the augmented models align with human diagnostic abilities under distribution shifts, we tested four trained radiologists on the out-of-distribution data (840 collected psychophysical trials in total) and compared their performance against the models. The involvement of medical professionals serves as a valuable benchmark for the models' diagnostic accuracy, allowing us to directly compare the effectiveness of DL-augmented interpretations with that of human experts when confronted with out-of-distribution data.

As a second contribution, we present *DreamOn,* a novel generative adversarial network (GAN) based data augmentation approach designed to enhance model robustness. GANs have previously gained recognition as a data augmentation strategy in various domains, especially in medical imaging [e.g., (25, 26)]. This has been motivated by a lack of available large, labeled training datasets for certain medical imaging modalities or specific medical conditions. However, in this study, we extend the traditional use of GANs by implementing a novel interpolation technique between classes, rather than simply generating synthetic samples. This was inspired by the process of dreaming in humans, where episodic memories are recombined to generate novel visual experiences during REM (Rapid Eye Movement) sleep [e.g., (27)]. We mimic this process by first teaching a GAN to create images of a single class. Once trained, we introduce a pair of classes to the Generator, with the classes being combined in varying proportions rather than being weighted equally. This prompts the Generator to synthesize images that blend characteristics from both classes. This interpolation process is crucial because it generates additional images that sit near the decision boundaries between classes, making these images more challenging to classify. Previous studies [e.g., (28)] have demonstrated that training a classifier on challenging images near decision boundaries can help the model establish more robust boundaries. This approach reduces the likelihood of overfitting to specific features and minimizes the influence of spurious correlations within the data. Consequently, this should help the model generalize better, particularly in high-noise environments, where maintaining performance is typically more difficult.

Aligning with this prediction, DreamOn-augmented datasets resulted in across the board substantial improvements in image classification accuracy under high-noise conditions as compared with other data augmentation strategies. While expert radiologist outperformed all models in high-noise settings, DreamOn augmentation helped to narrow the gap between expert radiologists and deep learning models when handling out-of-distribution data.

## 2 Materials and methods

The experimental design was structured to compare different off-the-shelf data augmentations and to test the hypothesis that DreamOn enhances model robustness compared to other data augmentation strategies. This was achieved by evaluating classification performance on the publicly available Breast Ultrasound Image Dataset [BUSI, see (29)], consisting of 780 labelled breast ultrasound images. As a comparison to DreamOn, we employed Manifold Mixup, Pixel-space Mixup, and more straightforward techniques such as rotation, flipping, and brightness & contrast changes. To assess the impact on the robustness of these augmentation techniques, we introduced three types of parameterized noise—Gaussian, speckle, and salt & pepper—each applied at seven intensity levels to get different test sets featuring a distribution shift. The different models were compared based on their ability to maintain high balanced accuracy and low expected calibration error (ECE) across noise levels. Additionally, the inclusion of the *DreamOff* control dataset allowed us to determine whether the observed improvements were due to the interpolation strategy used in DreamOn rather than just adding GAN-generated images to the training set. Lastly, four trained radiologists served as a benchmark by evaluating a subset of the test data, allowing us to put the model results into perspective. This comparison provided a clearer understanding of the deep learning models' robustness relative to human expertise, especially under high-noise conditions.

### 2.1 Off-the-shelf data augmentation

We implemented and evaluated three common data augmentation strategies known to enhance the robustness of DL classifiers. Firstly, in what we call *standard data augmentation* (SDA), we applied random rotation (–15° to +15°), random horizontal flip as well as random adjustments in brightness and contrast to training images, as reported to be among the most effective ones in medical imaging (21). Random rotations and horizontal flips were included to simulate variations in patient positioning and imaging angles. Brightness and contrast are parameters that depend on the patient and examined tissue, but they can also be adjusted by the physician to some extent on the ultrasound device and may vary between different devices. Note that vertical flips were not used here, as this would not have been consistent with the shape of ultrasound images (i.e., an increase in the field of view with increasing depth, displayed from top to bottom).

Secondly*, pixel-space Mixup* where training examples are created by linearly interpolating between random pairs of samples across classes on the pixel level and their corresponding labels (23). Lastly, *Manifold Mixup* extends pixel-space Mixup to the feature level, interpolating between representations at various latent layers of the network (24). Note that this was done during training and therefore with changing weights. The mixing proportions were determined by

λ(x,y)=λ⋅x+(1−λ)⋅y

where *λ* is a random value drawn from a Beta distribution *λ* ∼ Beta (*α*, *α*), *x* and *y* are two inputs.

### 2.2 DreamOn data augmentation

In addition to these off-the-shelf data augmentations, we evaluate a novel approach that combines the use of GANs to generate novel synthetic data with a biologically inspired idea: during REM sleep it is thought that previous episodic memories are recombined to internally generate novel visual experiences [e.g., see (27, 30)]. Here we mimic this process by feeding the generator of a fully trained conditional GAN with interpolated class labels and segmentation masks. To find out whether standard data augmentation can be combined with DreamOn to further improve the robustness, we also applied standard data augmentation (as described above) to the DreamOn images (*DreamOn* *+* *SDA*).

To implement DreamOn, we closely followed the approach proposed by Iqbal and Ali (31) where a GAN is trained on medical images. However, we augmented the method described by a conditional GAN model similar to Odena et al. (32), allowing input of the desired class label, so newly generated synthetic images preserve a given target class. This is because the dignity of ultrasound imagery is not solely conveyed by the mass shape but also by other factors. Providing the generator with class information therefore allowed the learning of such. Additionally, the segmentation mask that was fed to the generator was synthesized by a separate GAN trained only on the BUSI segmentation masks. This enabled the synthesis of interpolated segmentation masks.

**Figure 1:**
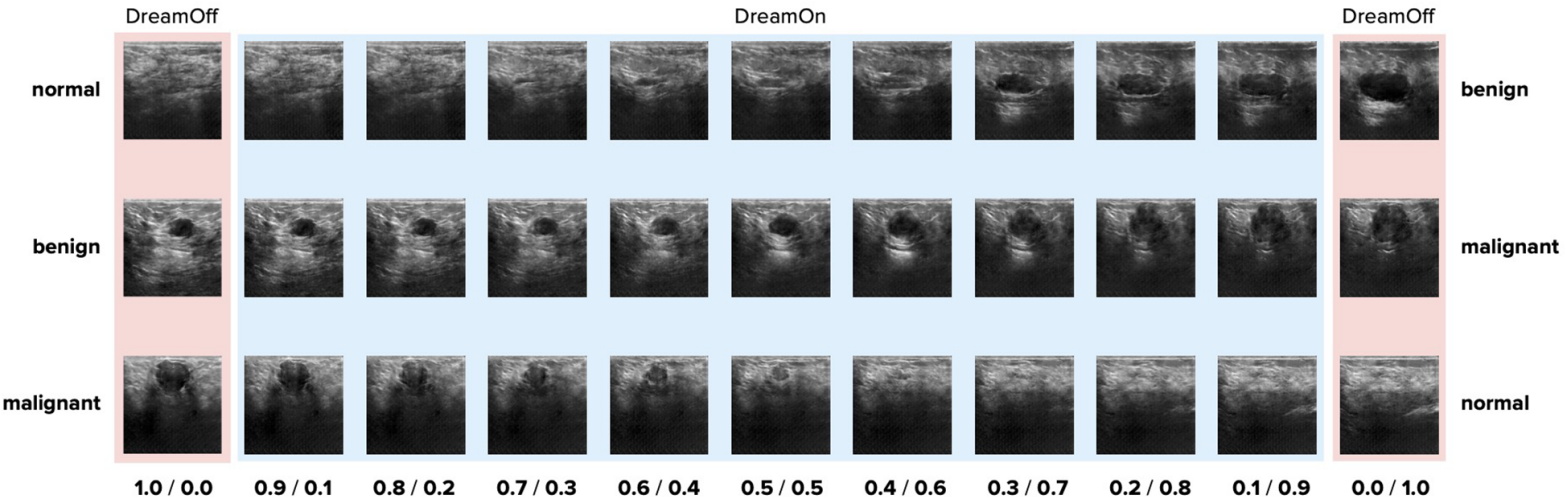
Example images generated by the proposed DreamOn data augmentation method. A sample interpolation for each pair of classes is shown in fractional steps where the weight of the third, unused class was set to zero. On each end, only one class was used as input (DreamOff). Upon close inspection, there are checkerboard artifacts present in the synthesized images. Such artifacts are common in images generated by convolutional neural networks [e.g., see (32)]. Note that the same artifacts are also found in images used for DreamOff (left side and right side).

**Figure 2:**
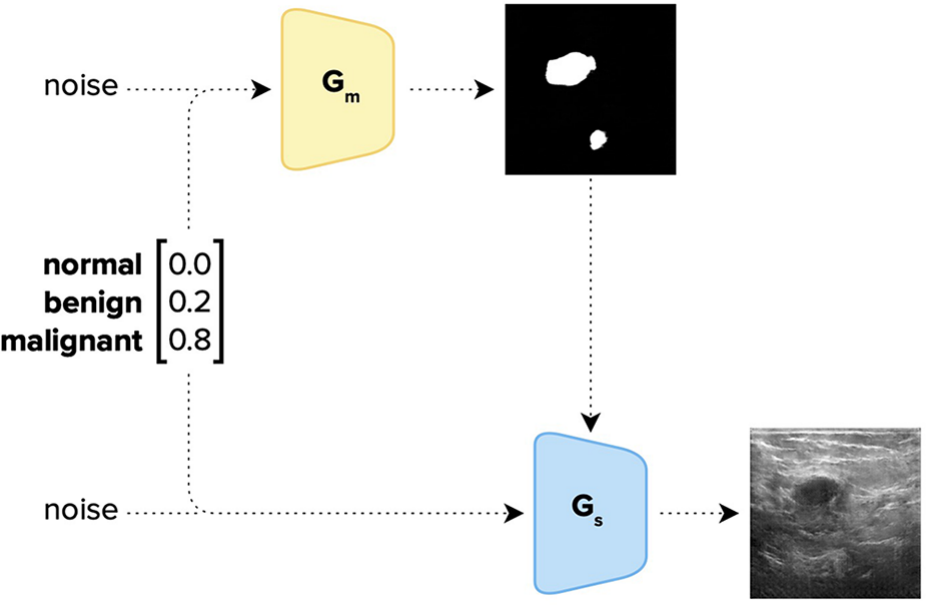
Image generation pipeline of DreamOn. The Generator *G*_m_ was trained on the segmentation masks of the BUSI dataset, and *G*_s_ both on the segmentation masks and the corresponding BUSI sonography images. The two GANs were trained separately and combined only afterward; *G*_m_ is used to generate a segmentation mask that is fed to the Generator *G*_s_ together with a class vector [1, 3] and a noise vector [1, 400] to generate a synthetic image characterizing the given mixture of class attributes (in this example: 0% normal, 20% benign and 80% malignant). The class vector was then set to be the ground-truth label of the generated image.

To generate interpolated images, two non-zero weights were assigned to two classes such that they sum up to 1. See Figure 1 for three examples. Since the classes of the BUSI dataset are not balanced, assigning uniformly random weights to classes when synthesizing DreamOn images could potentially lead to an unfair advantage compared to the other data augmentation methods. To account for this potential confounder, we constructed the image generation pipeline such that the average weight input per class over the whole DreamOn dataset matched the true proportions of the BUSI dataset (normal: 17%, benign: 56%, malignant: 27%). The ground truth label of the DreamOn dataset was identical to the two non-zero weights used for its generation, the third unused class was set to zero. The whole DreamOn pipeline is depicted in Figure 2. As it has been shown before, introducing such out-of-distribution (o.o.d.) data to training imagery can itself lead to improved robustness (33). To test whether a potential increase in robustness can be linked to interpolations rather than simply adding o.o.d. data, we employed an additional dataset of images created by the DreamOn architecture except for only using one class per image as input. We call this control data set *DreamOff*. For the detailed model architecture and training pipeline, see Figures S1 and S2 in the Supplementary Material. The code is available at https://github.com/lucle4/DreamOn.

### 2.3 Datasets

The BUSI dataset consists of 780 labelled (normal: 133, benign: 437, malignant: 210) images of breast ultrasound images, each with its corresponding segmentation mask (34). We randomly split the dataset into training (600), test (90), and validation (90) subsets. To enable maximal comparability between different data augmentation strategies, all training datasets consisted of two parts: first, the 600 original (non-augmented) images; and second, 600 augmented images which we manipulated/generated according to the respective approach (SDA, pixel-space Mixup, Manifold Mixup, DreamOn, DreamOn + SDA). Overall, we note that data augmentation approaches operating on the feature level, such as Manifold Mixup and DreamOn, can interpolate features at higher semantic levels of the information compared to pixel-wise data augmentation methods. For comparison, we also included a dataset that contains only original BUSI images (no data augmentation used; referred to hereafter as *Vanilla*). The composition of all training datasets is given in Table 1. In all datasets (including testing and validation), the class proportions were held constant (normal: 17%, benign: 56%, malignant: 27%).

**Table 1:** Composition of the different ResNet-18 training datasets.

Dataset	Composition	
DreamOn	600 BUSI images +	600 generated images (2 classes per image)
DreamOn + SDA		600 generated images (2 classes per image) with SDA
DreamOff (no interpolation)		600 generated images (1 class per image)
Manifold Mixup (24)		600 BUSI images with Manifold Mixup (*α* = 0.8)
Pixel-space Mixup (23)		600 BUSI images with pixel-space Mixup (α = 0.8)
Standard Data Augmentation		600 original images with standard data augmentation
Vanilla		No data augmentation

SDA, standard data augmentation.

Note that all models except the vanilla model were trained on 1,200 images in total (the original BUSI images plus an augmented set of 600 additional images).

*α* denotes the parameter of the Beta probability distribution that was used for Mixup. Note that usually, lower values of *α* yield better results [e.g., (24)]. We settled on a higher value since here, Mixup is implemented on only half of the dataset.

### 2.4 Test datasets

**Figure 3:**
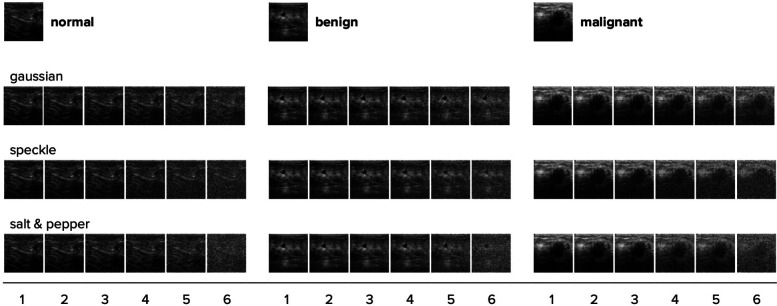
One randomly chosen test image for each category is shown with all three noise types and their intensity levels applied. With each step (1–6), the noise intensity doubles. The original image with no noise is shown on top.

To test model robustness, we created three different test datasets by applying different noise types—gaussian, speckle, and salt & pepper—each with six intensity levels to the test dataset. These noise types were specifically chosen because they are representative of common distortions encountered in ultrasound imaging. Gaussian noise simulates random fluctuations that can occur due to electronic interference, speckle noise reflects granular noise patterns typical in coherent imaging systems like ultrasound, and salt & pepper noise models impulse noise that can result from sudden disturbances or transmission errors. By using these noise types, our robustness evaluation is designed to closely mimic the challenges faced in real-world ultrasound imaging, ensuring that our model's performance is assessed under conditions that are likely to be encountered in practical scenarios [see (35)]. See Figure 3 for some examples. With each ascending level, there's a doubling in noise intensity, with the highest level calibrated such that most models perform at chance level (i.e., with ∼33% accuracy).

### 2.5 Classifier models

Each training dataset (see Table 1) was used to train a ResNet-18 model from scratch [for architectureal details, see (36)] in Pytorch (37). As has been shown before, ResNet-18 can be successfully used for classifying medical imagery (38). We used the Adam optimizer and cross-entropy loss with common hyperparameters (epochs = 100; batch size = 20; learning rate = 0.001; *β*_1,2_ = 0.9, 0.999) without any finetuning. Model parameters were initiated randomly. Each ResNet-18 was trained for five runs to account for random variations. The checkpoint that reached the highest balanced accuracy on the validation dataset was used for testing. Balanced accuracy, which is calculated as the average accuracy per class to account for class imbalance, serves as our primary metric for model performance. We report and compare the median balanced accuracy across the five training runs for each training strategy to draw our main conclusions. Training of classifiers as well as the GAN was performed on UBELIX (http://www.id.unibe.ch/hpc), the HPC cluster at the University of Bern using an NVIDIA A100 GPU. The code for the different training strategies is available at https://github.com/lucle4/DreamOn.

### 2.6 Human observers

To benchmark the performance of the different models against human experts, we presented noisy images to *n* = 4 trained radiologists from the University Hospital of Bern. Of the participating radiologists, 2 were female and 2 were male, with a median experience of 18 years (*SDexp* = 15.1). In a forced-choice image classification task, they had to classify 210 Gaussian noise images (30 images per noise level). Gaussian noise was used for testing due to its standard use in assessing robustness, therefore providing a reliable benchmark for comparing the performance of deep learning models and human experts in a controlled environment [e.g., (33)].

### 2.7 Performance metrics

We assessed model robustness using two main performance metrics: *balanced accuracy* and *expected calibration error* (ECE). Balanced accuracy is defined as the mean over the average accuracy per class, accounting for class imbalance in the dataset. It is a suitable metric for our study because it evaluates model performance across all classes, ensuring that improvements in robustness are not biased by the predominant class. The ECE measures the difference between the predicted confidence levels and the actual outcomes, providing insight into how well-calibrated the model's predictions are. Well-calibrated predictions indicate that the model's confidence aligns with its accuracy, an important factor in medical imaging where decision-making should reflect a reliable estimation of uncertainty. Both metrics were used to assess the stability of model performance under various noise levels, which serve as a proxy of robustness against real-world image distortions.

To establish a threshold above which model performance could be considered significantly better than chance, we used the Clopper-Pearson method to calculate the upper bound of the 95% confidence interval around chance-level accuracy. For each noise condition, we compared the model's balanced accuracy against this threshold, considering performance significant if it exceeded this value.

## 3 Results

**Figure 4:**
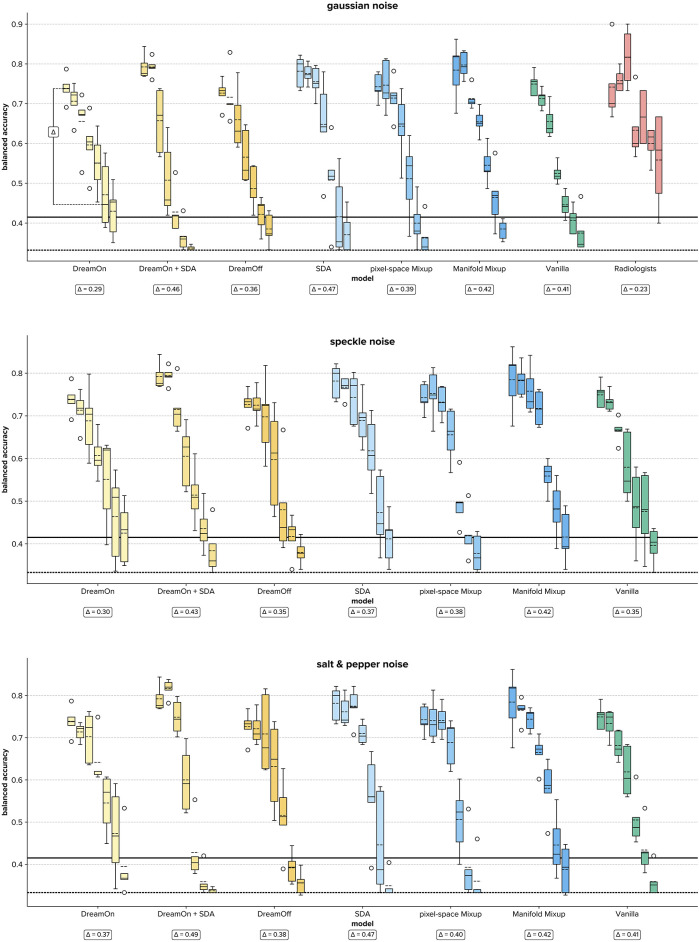
Boxplots of balanced accuracies for each data augmentation strategy (different colors) per noise level (0–6, boxes with identical colors) for all three noise types (different panels). Each box summarizes the test results of five training runs (radiologists: *n* = 4). Wrt. the boxes, the dotted line depicts the mean, and the straight line the median. Note that radiologists only rated images with Gaussian noise. The *Δ* annotations depict the difference between the highest and lowest median accuracy overall noise levels, with lower *Δ* values indicating a more stable model. A visual example to clarify the *Δ* value is given for DreamOn in the Gaussian noise condition. Bold horizontal lines indicate chance performance (dotted) and the upper bound of the 95% confidence interval (solid), i.e., all median and mean values above the horizontal bold solid line indicate classification performance that is significantly above chance. SDA, standard data augmentation.

Across all three noise types and for all models, the balanced accuracy decreases as a function of noise intensity (Figure 4). However, this was not the case for the radiologists, for whom the performance increased from noise levels 1–3. Looking at the results in more detail, several patterns emerged. First, on original images (i.e., no noise), all DL models outperformed radiologists in terms of their median accuracy, indicating that in an environment with no added noise, model predictions are more accurate than human judgments. In this setting (original images), the Manifold Mixup and standard data augmentation outperform the other data augmentation strategies as well as the vanilla model. Second, in the low noise regime (level 1–3), Mixup approaches as well as standard data augmentation approaches continue to dominate—outperforming DreamOn and the vanilla model as well as radiologists. Third, in the high noise regime (level 4–6) however, the tables turn: here, radiologists outperform all DL models, indicating a robustness gap between human experts and models.

Compared to all other evaluated data augmentation approaches, DreamOn features the highest median balanced accuracy in the high noise regime (best performing in 6 out of 9 high noise levels, see Table 2), thereby reducing the robustness gap between human observers and models. Notably, there is a clear superiority of DreamOn compared to DreamOff. It can, therefore, be safely argued that it is the interpolation that led to better performance rather than the introduction of GAN artifacts and therefore merely o.o.d. data. Interestingly, adding SDA to DreamOn images does not lead to further improvement in robustness. Quite in contrary, for high noise levels, this model performs among the worst.

**Table 2:** Median balanced accuracies (over five runs) in high-noise settings (levels 4–6).

		DreamOn	DreamOff	DreamOn + SDA	SDA	Pixel-space Mixup	Manifold Mixup	Vanilla	Radio logists
Gaussian	4	**0.551** *	0.464*	0.418	0.518*	0.544*	0.531*	0.442*	0.667*
	5	0.447*	0.444*	0.360	0.353	0.380	**0.469** *	0.413*	0.617*
	6	**0.453** *	0.373	0.333	0.333	0.340	0.400	0.347	0.584*
Speckle	4	**0.620** *	0.438*	0.509*	0.607*	0.496*	0.569*	0.487*	
	5	**0.509** *	0.433*	0.424*	0.447*	0.400	0.482*	0.480*	
	6	**0.433** *	0.378	0.360	0.431*	0.367	0.393	0.404	
Salt & Pepper	4	0.571*	0.513*	0.404	0.560*	0.524*	**0.587** *	0.487*	
	5	**0.467** *	0.393	0.347	0.387	0.373	0.424*	0.427*	
	6	0.367	0.356	0.333	0.333	0.333	**0.393**	0.351	

For each noise level, the highest accuracy is displayed in bold text. In six out of nine cases, DreamOn performs best (excluding radiologists), in the other cases, it performs second best. DreamOn performs in all high noise settings except one (eight out of nine) significantly above chance.

* Indicate classification performance that is significantly above chance performance.

While DreamOn may not achieve the highest accuracy in low-noise and no-noise conditions, it exhibits the greatest robustness against noise, with the lowest drop in accuracy as noise levels increase, and consistently outperforms other methods in the high noise regime, where maintaining stable performance is crucial for real-world medical imaging applications.

Additionally, we were interested in determining the extent to which models can sustain a performance significantly above chance under increasing levels of noise. Treating single image classification trials as independent Bernoulli trials, we calculated binomial 95% confidence intervals using the Clopper-Pearson method (39). This statistical approach enables us to establish the minimum performance threshold above which models can be considered to significantly exceed chance performance. For a chance level of p=1/3 (depicted as dotted horizontal lines in Figure 4), and *n* *=* 90 classification trials (corresponding to the size of the test dataset), the upper bound of the one-tailed 95% confidence interval is ∼0.411 (depicted as solid horizontal lines in Figure 4). Comparing median model performances with this threshold, we find that DreamOn performs significantly above chance for all but one (salt and pepper level 6) noise levels (see Table 1). Remarkably, under extreme noise conditions (noise level 6), no other model surpassed the chance level threshold, with the sole exception of the SDA model. However, it is important to note that the SDA model's performance did not consistently exceed chance across most other high noise conditions.

To further quantify robustness, we calculated the difference between the highest and lowest reached median balanced accuracy for each model (Δ, Figure 4). This metric provides a direct quantification of how consistent a model's performance is across varying datasets. A smaller difference indicates that the model maintains its accuracy level regardless of changes in the data, signifying higher stability. When comparing this relative drop in median accuracy (*Δ*) across data augmentation strategies, we find that DreamOn features the lowest difference irrespective of the noise type, and thus shows the most stable performance. This lines up with the radiologists, who show an even lower delta in the gaussian noise condition.

When examining the ECE, we find a similar pattern as with the balanced accuracy (see Figure S3 in the Supplementary Materials). Across all noise types and models, the ECE increases with increasing noise intensity, indicating reduced model calibration as a function of noise intensity. In practical terms, this means that under higher noise levels, the confidence scores provided by the models do not reliably reflect the true probability of a correct prediction, mostly leading to overconfident classifications. While model calibration generally declines in high-noise settings, DreamOn produces comparably well-calibrated probability estimates, with confidence levels that closely align with actual prediction accuracy even under noise. Only Manifold Mixup performs similarly in these challenging conditions. Taken together, maintaining above-chance performance in high-noise settings and preserving calibration indicate that DreamOn enhances the model's ability to make accurate predictions with reliable confidence estimates even under distribution shifts.

### 3.1 Consistency among radiologists

To investigate the inter-rater reliability of the radiologists, we calculated the Fleiss' Kappa (40). For noise levels 0–4, *κ* was between 0.544 (noise level 4) and 0.681 (noise level 2) per level, corresponding to moderate up to substantial agreement. For noise levels 5 and 6, *κ* was 0.464 and 0.380, corresponding to fair up to moderate agreement (41). Thus, this consistency analysis indicates that the agreement among radiologists generally decreases as a function of noise intensity. This pattern suggests that even experienced professionals can struggle to maintain diagnostic accuracy. The observed variability among human raters can result from factors such as the complexity of certain images, the potential for increased subjective interpretation, and the noise's impact on key features critical for diagnosis. Nevertheless, in high-noise scenarios, even the worst-performing radiologist performs better than all DL models evaluated in this study. This indicates that while DreamOn effectively narrows the robustness gap between expert radiologists and deep learning models, the remaining gap is not a mere product of differences among radiologists but highlights the fundamental challenges in replicating human diagnostic resilience in adverse conditions.

## 4 Discussion

We conducted a comprehensive investigation of different popular data augmentation strategies on the robustness of a ResNet-18 model trained to classify breast ultrasound images. We also compared the model's performance with human experts in the field. Our results indicate that DreamOn—our proposed GAN-based data augmentation method that generates REM-dream-inspired synthetic data—can notably improve the model's robustness, thus narrowing the gap between human observers and DL models in the high noise regime.

While all models experienced a decline in accuracy with increasing noise, DreamOn consistently outperformed other methods in the most challenging noise settings, demonstrating a notable improvement in robustness compared to standard approaches. It was the only method that maintained performance significantly above chance across nearly all noise levels. This robustness, coupled with its stability (evidenced by the smallest decrease in performance from no-noise to high-noise conditions, *Δ*), positions DreamOn as a well-suited strategy for enhancing deep learning models in noise-intense medical image analysis.

However, despite DreamOn's robust performance in high-noise environments, we observed a drop in accuracy in low-noise regimes. This reduction in performance could be attributed to the introduction of unnecessary complexity, where the challenging interpolations generated by DreamOn might lead the model to overfit on ambiguous examples rather than optimizing for cleaner, more straightforward cases. In such settings, the model could become overly specialized in handling difficult scenarios, resulting in a trade-off where robustness in high-noise environments comes at the expense of accuracy in low-noise or clean data conditions.

Although DreamOn's performance in low-noise and no-noise settings is not as strong as some other augmentation methods, this should be viewed in the context of real-world medical imaging scenarios, where noise is often unavoidable. A model that excels in clean environments but rapidly deteriorates under noisy conditions may not be as useful in practice. DreamOn's strength lies in its ability to maintain accuracy as noise levels increase, exhibiting the lowest drop in performance across varying noise intensities. This robustness is critical in medical image analysis, where the ability to produce reliable results under suboptimal conditions is often more valuable than peak performance in ideal scenarios. Therefore, DreamOn's superior performance in high-noise environments suggests it is a more reliable choice for applications where image quality cannot always be guaranteed.

Additionally, when combining DreamOn with Standard Data Augmentation (SDA), we noticed a performance drop compared to using either strategy alone. This may be due to conflicting learning signals: while DreamOn encourages the development of robust decision boundaries by creating difficult, boundary-challenging cases, SDA introduces broader variability through transformations like rotations and flips, which do not necessarily increase difficulty. The model might struggle to reconcile these different types of data, leading to suboptimal performance when both strategies are employed together. These observations highlight the complex interactions between different data augmentation techniques and underscore the need for further investigation into their combined effects.

Furthermore, the superior robustness of DreamOn compared to other data augmentation methods highlights the potential of GAN-based techniques in enhancing the generalization capabilities of deep learning models in medical imaging [for a review, see (42)]. The interpolation of class labels and segmentation masks enables the model to learn from a range of image variations not provided by traditional augmentation methods. The improvement in model robustness indicates that DreamOn could assist in preparing models to manage the inconsistencies and variability found in clinical settings. This enhanced robustness in high-noise environments suggests that such AI-driven tools could be particularly valuable as complementary aids to radiologists. By integrating models like DreamOn into diagnostic workflows, it is possible to develop AI systems that can assist in analyzing challenging cases where image quality is compromised, thereby enhancing the overall diagnostic accuracy and confidence of radiologists. However, it is important to note that it is uncertain how well the findings related to the employed noise types can be generalized to real-world noise stemming from different imaging equipment and protocols, or patient-specific factors such as movements or biological variability (e.g., tissue density).

While radiologists outperformed all models at higher noise levels, this emphasizes the ongoing importance of human expertise in medical image analysis. However, the lower accuracy of radiologists on original images without added noise perturbations might reflect the model's ability to detect subtle patterns not readily apparent to the (trained) human eye.

We also note that, similarly to REM dreams, the semantic meaning of produced interpolations might not directly correlate with reality. This is because diagnostic work-up is done along the lines of specific guidelines that assign findings to discrete categories. There are benign lesions that mimic malignancy and vice versa, and some lesions indeed have an intermediate appearance between malignant and benign (what ultimately makes them suspicious). But there is no continuum between these categories (43) such as is the case with DreamOn. Nonetheless, such augmented samples help in enhancing model robustness and act as an effective regularization component (21, 44, 45). We advocate that for clinical setups, while accuracy is important for deep learning models, their robustness and reliability might be even more important to ensure time-effective and trustworthy human-in-the-loop AI-assisted clinical workflows. In this regard, the proposed DreamOn data augmentation proposes a promising starting point to develop a stable framework for clinical situations where suboptimal imaging conditions occur.

### 4.1 Limitations and future research

In the present study, we only investigated the robustness of one DL architecture (ResNet-18) and only employed a single medical dataset. Even though clinically relevant, the BUSI dataset is relatively small (780 unique images). Future research should thus focus on employing the DreamOn augmentation strategy for a wider variety of DL models, medical datasets, and additional types of perturbations to assess its robustness across more varied and complex noise conditions. It is also important to note that other advanced data augmentation strategies, such as additional GAN-based methods [e.g., (46)], further Mixup variants [e.g., (47)], and data augmentation with transformers [e.g., (48)], were not covered in this study. Future research should explore these strategies to further validate and potentially enhance the robustness of our approach. Furthermore, the DreamOn approach could be improved by integrating other generative approaches such as diffusion models (49). Additionally, it would be ideal to develop a model that not only exhibits increased robustness in high-noise regimes but also maintains high accuracy across the board, including in low-noise and no-noise conditions.

One limitation worth noting is that radiologists' data was exclusively obtained for gaussian noise, with other noise types not being covered. Nevertheless, it is known that humans typically perform well across different noise types in image classification tasks [e.g., (10)]. Therefore, we anticipate that the radiologists' performance on the additional noise types would be similar to their performance on gaussian noise.

## 5 Conclusion

In conclusion, the present study illustrates that REM-dream-inspired conditional GAN-based data augmentation through class and segmentation mask interpolation presents a promising approach to enhancing the robustness of deep learning models against noise perturbations in medical imaging. By benchmarking different data augmentation strategies against expert radiologists on out-of-distribution data, our study reveals a persistent gap in robustness between models and human experts, underscoring the need for continued advancements in AI to match human diagnostic proficiency. As the field continues to advance, incorporating biologically inspired data augmentation strategies could play a significant role in supporting radiologists and improving diagnostic accuracy in clinical settings.

## Data Availability

The original contributions presented in the study are included in the article/Supplementary Material, further inquiries can be directed to the corresponding author.
