# Supplementary material for: DreamOn: a data augmentation strategy to narrow the robustness gap between expert radiologists and deep learning classifiers
Source: Front Radiol. 2024 Dec 19;4:1420545. doi: 10.3389/fradi.2024.1420545 (PMC11696537; doi:10.3389/fradi.2024.1420545)
Supplement: Supplementary file 1 [file Datasheet1.pdf]

Supplementary Material

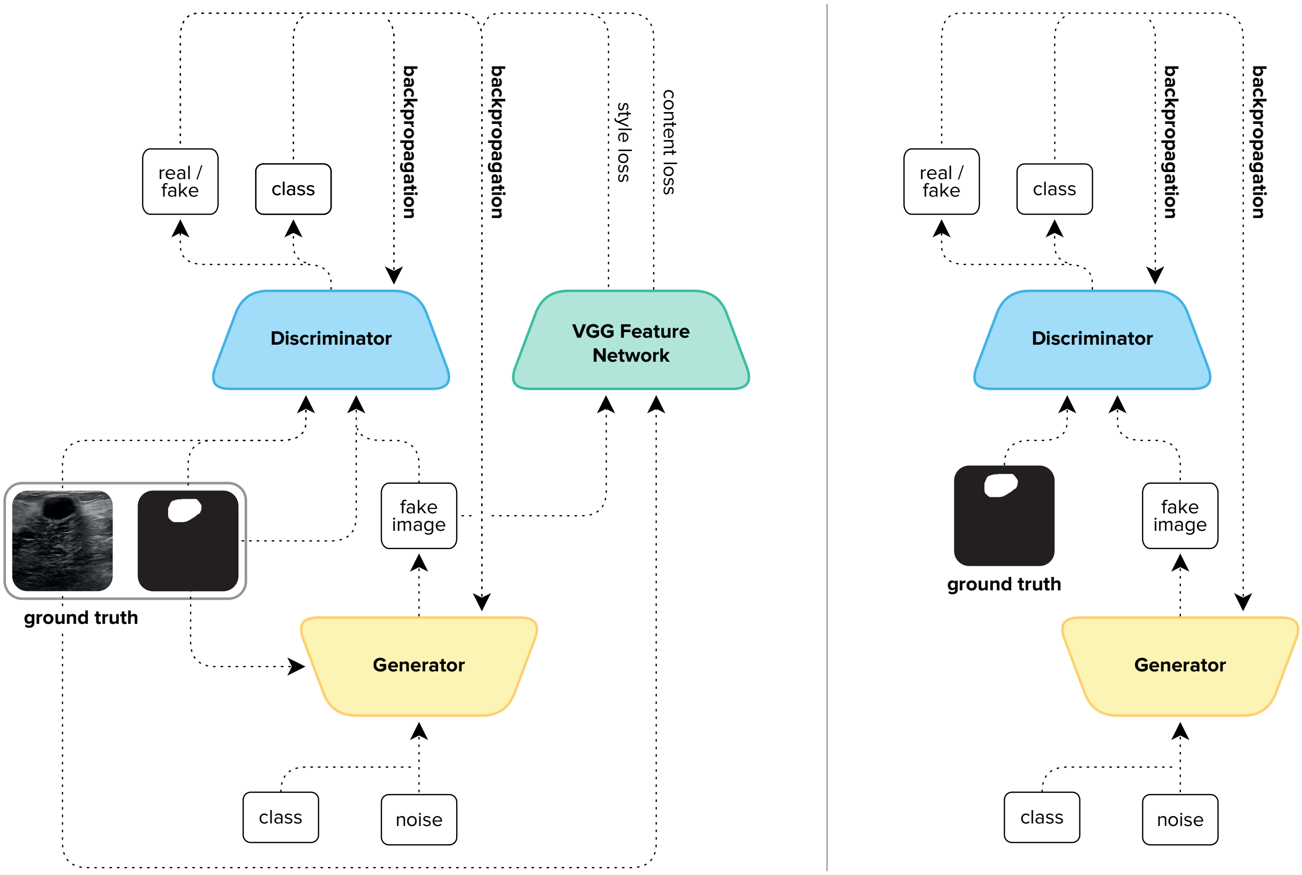

Supplementary Figure 1. DreamOn training pipeline. A subset of 600 images of the BUSI dataset (780 images in total) is used for training. The Adam optimizer is used for stochastic gradient descent. Both the Generator (G) and the Discriminator (D) aim to minimize the class loss. Conversely, G aims to maximize the adversarial loss (real/fake) whereas D aims to minimize it. For training, we chose the following set of hyperparameters: image size = 256 x 256; number of classes = 3; color channels = 1; epochs = 3,500; batch size = 10; noise vector size = 400; learning rate = 0.0002; $\beta_1 = 0.9$; $\beta_2 = 0.999$. (**A**) *left* G takes a noise vector, a class vector, and a segmentation image corresponding to the current class as input. Losses are calculated based on D (class loss and adversarial loss) and a pre-trained VGG-19 network that calculates an additional style and feature loss (for details, see Gatys et al., 2015; Iqbal & Ali, 2018). (**B**) *right* A simplified approach is used for the GAN creating artificial segmentation masks where only a class vector and a noise vector are used as input for G and the losses are only calculated based on the output of D. After training, the images created by G are fed into G of **A**. See **Figure 1** for the inference pipeline.

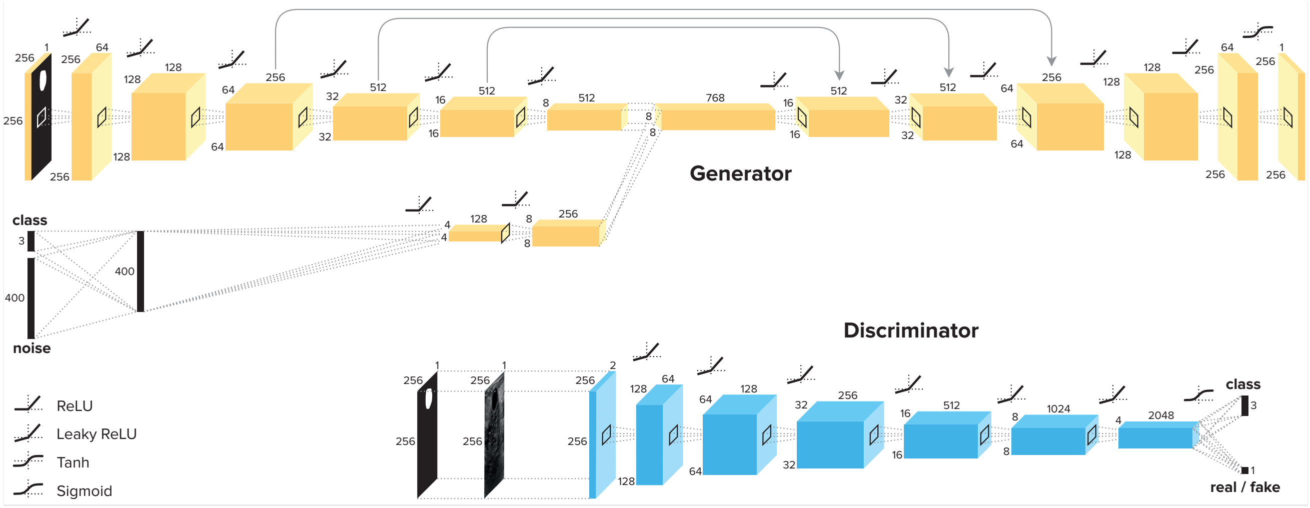

Supplementary Figure 2. Detailed model architecture of the DreamOn GAN (as in **Figure S1 A**) adapted from Iqbal & Ali (2018). Numbers denote the layer dimensions, and the respective activation function is shown above each layer. The kernel size equals 4 and is held constant throughout all layers. Arrows denote skip connections. Fully connected layers are depicted as black rectangles.

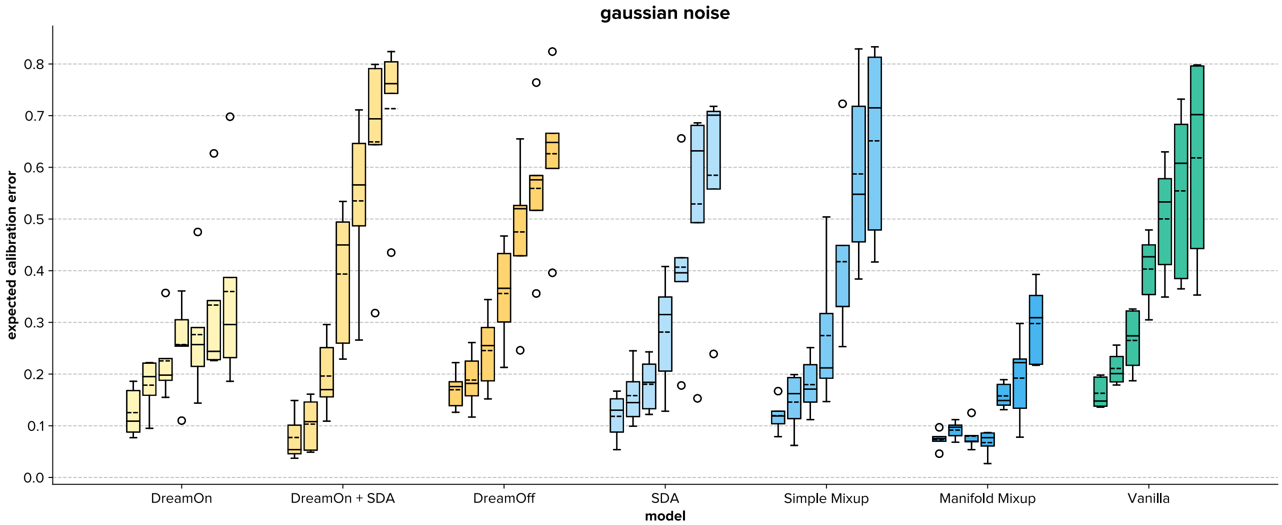
gaussian noise
expected calibration error
0.0
0.1
0.2
0.3
0.4
0.5
0.6
0.7
0.8
DreamOn
DreamOn + SDA
DreamOff
SDA
Simple Mixup
Manifold Mixup
Vanilla
model

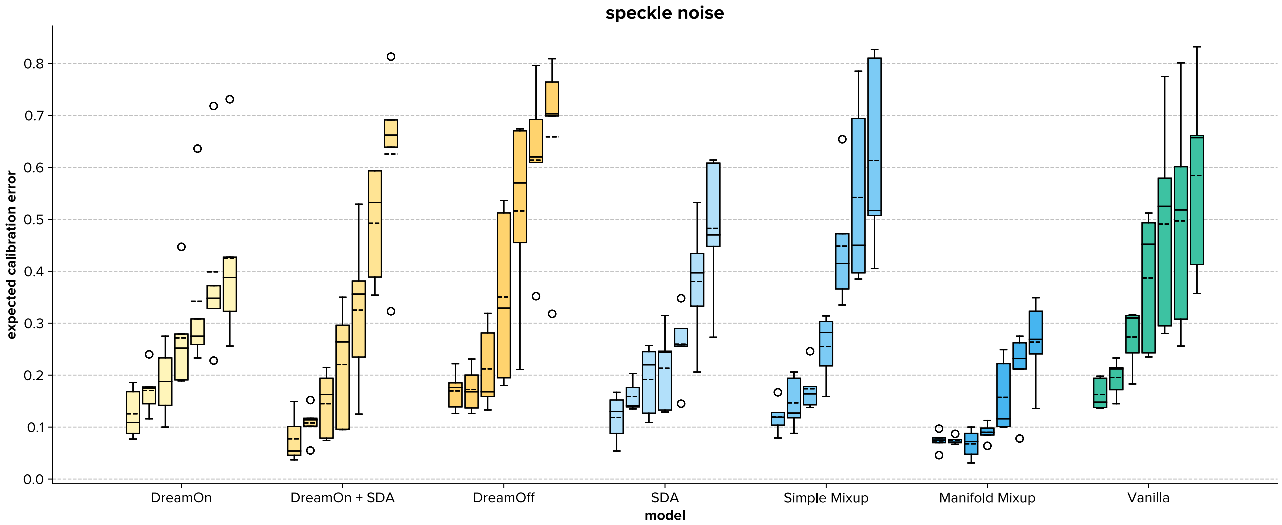
speckle noise
expected calibration error
0.0
0.1
0.2
0.3
0.4
0.5
0.6
0.7
0.8
DreamOn
DreamOn + SDA
DreamOff
SDA
Simple Mixup
Manifold Mixup
Vanilla
model

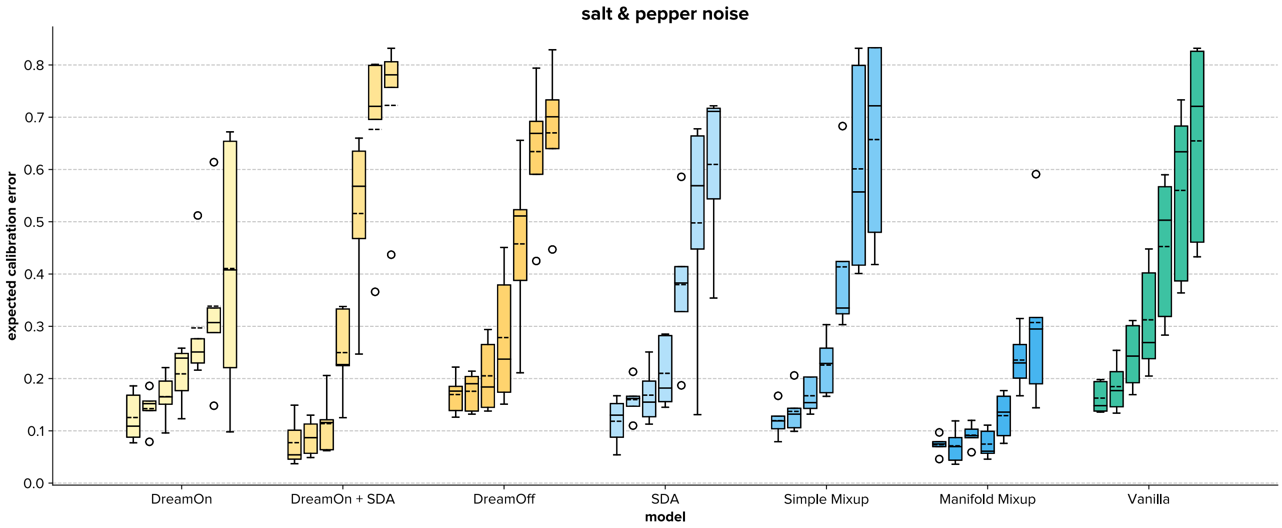
salt & pepper noise
expected calibration error
0.0
0.1
0.2
0.3
0.4
0.5
0.6
0.7
0.8
DreamOn
DreamOn + SDA
DreamOff
SDA
Simple Mixup
Manifold Mixup
Vanilla
model

Supplementary Figure 3. Boxplots of the expected calibration error (ECE) for each data augmentation strategy (different colors) per noise level (0 – 6, boxes with identical colors) for all three noise types (different panels). Each box summarizes the test results of five training runs. ECE is a metric used to assess how well the predicted probabilities from a classifier align with the actual outcomes (ground truth labels). The ECE calculation involves grouping the predicted probabilities into several bins (here, we used 10 equally spaced bins). For each bin, the model's average predicted probability (confidence) is compared with the actual accuracy (proportion of correct predictions). The absolute difference between these two values is computed for each bin. ECE is then the weighted average of these differences across all bins, with weights corresponding to the number of samples in each bin. A low ECE indicates that the model's predicted probabilities are well-calibrated, meaning the confidence level closely matches the actual accuracy. Conversely, a high ECE suggests that the model's probabilities are poorly calibrated, potentially being overconfident or underconfident in its predictions.

DreamOn consistently demonstrates lower ECE compared to most other strategies across all noise types (gaussian, speckle, and salt & pepper), indicating that it produces well-calibrated probability estimates where the model's confidence closely aligns with actual accuracy. Even as noise levels increase, DreamOn maintains relatively low ECE values, outperforming most other data augmentation strategies, with Manifold Mixup being the only strategy that performs comparably or better under certain noise conditions. In contrast, the DreamOn + SDA strategy generally shows higher ECE values compared to the standalone DreamOn strategy across all noise types. This suggests that combining DreamOn with Standard Data Augmentation (SDA) may lead to less well-calibrated probability estimates, as indicated by the higher ECE, potentially reducing the reliability of the model's confidence in its predictions.
